# The Relationship between Spiritual Well-Being and Resilience in Patients with Psoriasis

**DOI:** 10.1155/2021/8852730

**Published:** 2021-03-26

**Authors:** Mohammadhossein RahimZahedi, Camellia Torabizadeh, Majid Najafi Kalyani, Seyed Alireza Moayedi

**Affiliations:** ^1^Student Research Committee, Shiraz University of Medical Sciences, Shiraz, Iran; ^2^Department of Nursing, Shiraz University of Medical Sciences, Shiraz, Iran

## Abstract

Psoriasis skin disease affects the patients' health and quality of life to a great extent. Given the chronic nature of the disease, identifying the factors affecting adaptation to the disease can provide guidelines required for helping these patients deal with their problems. This study was conducted with the purpose of investigating the relationship between spiritual well-being and resilience in patients suffering from psoriasis. The present study is a descriptive-analytical work conducted in the largest city in the south of Iran in 2019. 150 patients diagnosed with psoriasis completed Ellison and Paloutzian's Spiritual Well-Being Scale and Connor and Davidson's Resiliency Scale. Data were analyzed using SPSS v. 20, descriptive (frequency distribution, mean, and standard deviation) and inferential statistics (Pearson, regression, and *t*-test). The significance level was set at 0.05. The obtained mean scores were 54.84 ± 13.25 for resilience and 73.22 ± 11.13 for spiritual health. Spiritual health predicted 43% of the variance of resilience, and all resilience-related factors had a significant positive relationship with spiritual well-being-related factors (*P* > 0.05). An analysis of the relationship between demographic variables on the one hand and resilience and spiritual well-being on the other indicated that an increase in the patients' academic status, duration of the disease, and age correlated with an increase in their resilience and spiritual well-being. Also, male patients and married patients were found to possess higher levels of resilience and spiritual well-being. According to the findings of the present study, spiritual well-being correlates with resilience in patients with psoriasis. Considering the chronic nature of the disease, it is recommended that more attention be paid to promoting spiritual health in the care plans of these patients.

## 1. Introduction

Psoriasis is a chronic, inflammatory, non-contagious, immune-mediated dermatosis [1, 2]. The prevalence of psoriasis is estimated to be 1.5% to 11.43% in various countries [3, 4]; it is reported to be less than 0.5% by Asiatic studies [5–7]. The main cause of psoriasis is unknown, but the disease is affected by a variety of contributory factors including ethnic, geographical, genetic, and environmental factors [8, 9]. Stress has been reported as one of the principal environmental risk factors of the disease [10]. On the other hand, poor self-image [11] and the unpredictable and incurable nature of the disease [12] cause significant mental pressure and impose great social, as well as individual, costs on patients [13]. The dramatic changes and the progression and regression of the disease have been associated with extreme tension in patients with psoriasis [14]. As a result, the disease tends to progress in different stages of the patients' life and consequently decreases both the patient's quality of life and response to treatment [15, 16].

Analyses of the biological changes in patients diagnosed with psoriasis show similarities between the mechanism of these physical changes and the ones detected in individuals affected by anxiety [17]. Chronic skin diseases, such as psoriasis, which affect an individual's appearance adversely affect the patient's self-esteem [11] and religious faith [18] and disrupt the patient's adaptation mechanisms and interpersonal communications due to a lack of hope for the future, and eventually lead to a spiritual crisis [19]. Spiritual confusion greatly reduces interest in treatment [20], whereas spirituality improves the patient's quality of life by giving purpose and meaning to his/her life [21]. Many factors which can help patients deal with the challenges raised by chronic diseases have been proposed, spiritual well-being being one of the most significant [22, 23].

Spiritual well-being has been defined as stability in life, inner peace, harmony, and solidarity, and a close relationship with oneself, the Creator, the society, and the environment [24, 25]. There are two dimensions to spiritual well-being: religious well-being and existential well-being. Religious well-being is related to a person's relationship with God and religion, while existential well-being pertains to an individual's subjective well-being with regard to the meaning and purpose of life and satisfaction with life [26]. Spiritual sources are perceived as protective agents against diseases and stress. In various studies, it has been demonstrated that fortifying the spiritual dimension of life can alleviate the symptoms of diseases [27], despair and depression [28], and improve one's adaptation ability [29], mental health [30], social function [31], quality of life [32], meaning of life [27], and attitude towards life [33].

Although physical and psychological problems are more prevalent among patients with psoriasis than others [34], some patients can easily adapt to the severe problems pertaining to the disease and live normal lives thanks to certain personality traits, including resilience [35]. The positive influence of resilience in ameliorating depression [36], improving quality of life [37], and adaptation to stressful conditions, particularly for patients suffering from chronic diseases, has been proved [38]. Conner and Davidson have defined resilience as an individual's ability to create mental and spiritual balance in order to cope with life-threatening situations [39]. Studies also indicate that stronger resilience in patients diagnosed with chronic diseases leads to an increase in their acceptance, flexibility, coping ability, and mental health [40].

In spite of the significant prevalence and incurability of psoriasis, its relation to quality of life and psychological problems, such as depression and anxiety, has been analyzed in only a few studies [41, 42]. Designing appropriate interventions to improve the psychological conditions of patients with psoriasis requires preliminary analyses of the effective and protective factors in this disease. Although resilience and spirituality have always been considered as protective factors against psychological disorders caused by diseases, the researchers did not find any studies on the role of resilience and spirituality in patients diagnosed with psoriasis. Accordingly, the present study was conducted to explore the relationship between spiritual well-being and resilience in patients with psoriasis.

## 2. Materials and Methods

This is a descriptive-analytical study conducted on patients with psoriasis in Shiraz between May and October, 2019. The study population consisted of all of the patients with psoriasis who visited the dermatological centers in Shahid Faghihi Hospital, Motahari Clinic, and Imam Reza Clinic in the duration of the study. The participants were selected according to the purposeful sampling method and the inclusion and exclusion criteria of the study. The inclusion criteria were as follows: a definite diagnosis of the disease in question by a dermatologist; having had the disease for at least a year; willingness to participate in the study; being aged between 18 and 65 years; being able to speak and understand Farsi; a minimum of secondary school education; and being a Muslim. The exclusion criteria were as follows: suffering from another chronic disease, including diabetes, hypertension, or cancer; having a history of psychological disorders; having a history of suicidal attempts, use of psychotropic drugs, or hospitalization in a mental hospital; and having experienced a traumatic event in the past 6 months, e.g., losing a close relative or bankruptcy. Sample size was determined using Cochran's sample size formula for a correlational study:

(1) $$\begin{array}{c} N=\frac{\left(Z1+Z2\right)2^{*}{\left(1-r\right)}^{2}}{r^{2}}. \end{array}$$

Here, *Z*1 = a confidence coefficient of 95% equivalent to 1.96 and *Z*2 = a power of test coefficient of 80% equivalent to 0.84.

An estimation of the correlation coefficient between the subjects' spiritual well-being mean score and resilience mean score, *r*, is found to be 0.76 in a study [43]. The calculated sample size was 75 subjects, whose number was raised to 150 to decrease the probability of errors and in view of the possibility that some questionnaires were faulty. The data collection instruments consisted of a questionnaire related to the subjects' demographics (age, gender, marital status, education, occupation, and length of having had the disease) in addition to Ellison and Paloutzian's Spiritual Well-Being Scale and Connor-Davidson Resilience Scale.

Developed by Ellison and Paloutzian's in 1982 in the US, the Spiritual Well-Being Scale (SWBS) consists of 20 items, 10 of which measure existential well-being and the other 10 measure religious well-being. The score range is between 20 and 120. Answers follow a 6-point Likert scale, ranging from “totally agree” to “totally disagree” [44]. Ellison and Paloutzian reported the Cronbach's alphas of religious well-being, existential well-being, and the total Cronbach's alpha of the scale to be 0.91, 0.91, and 0.93, respectively [45]. The Cronbach's alpha of the Farsi version of the scale in Iran has been reported to be 0.82; the face validity and content validity of the scale have been tested and verified [46].

Connor–Davidson Resilience Scale (CD-RISC) (2003), developed in the US, consists of 25 items which address five different domains: personal competence/tenacity, trust in one's instincts/tolerance of negative affect, positive acceptance of change/secure relationships, control, and spiritual influences. The scale relies on a 5-point Likert scale, ranging from “absolutely incorrect” to “absolutely correct.” The score range is from 0 to 100, with higher scores indicating greater resilience. The creators of the instrument have tested its validity (via factor analysis and convergent and divergent validity tests) and reliability (via retest and came up with a Cronbach's alpha of 0.93) [39]. CD-RISC has been translated and normalized in Iran—the validity of the scale is tested and verified through factor analysis, and the reliability coefficient tests yield a Cronbach's alpha of 0.87 [47].

Data were collected by one of the researchers who visited the clinics where patients with psoriasis received treatment. For ethical considerations in research, in the first session, the researcher informed the patients about the objectives of the study and assured them that their information would be confidential. After the patients signed the informed consent form, they completed the self-administered questionnaire in the presence of the researcher. Prior to the study, necessary permissions were obtained from the research context. This study was approved by the Ethics Committee of Shiraz University of Medical Sciences (code: IR. SUMS.REC.1398.301).

After collection, the data were entered into SPSS *v*. 20. The variables and personal characteristics were described using descriptive statistics, including frequency percentage, mean, and standard deviation. The hypotheses were tested via the statistical methods of Pearson's correlation and stepwise regression. *t*-test was applied for comparing the spiritual well-being and resilience mean scores according to the demographic variables. Level of significance was set at *P* > 0.05.

## 3. Results

In the present study, the majority of the participants were married (56%) and aged between 18 and 52 years. 50% of the participants were male and 50% were female. Moreover, the disease duration of most of the participants (45.3%) was under 5 years (Table 1).

The results of the Kolmogorov–Smirnov test showed that the distribution of the data was normal.

Regarding the effects of the length of having the disease, the results of Tukey's range test showed that there was a significant difference between the spiritual well-being and resilience levels of the patients who had had psoriasis for over 7 years and the patients who had had the disease for less than 4 years or between 4 and 7 years (*P* < 0.05). The mean scores showed that an increase in the length of having the disease correlates with higher levels of spiritual well-being and resilience.

The results of Tukey's test showed significant differences between the age groups (under 30, 30–40, and over 40 years old) in terms of spiritual well-being and resilience (*P* < 0.05). According to the participants' mean scores, older patients possess more spiritual well-being and resilience.

Regarding the effects of education, the results of Tukey's range test showed that there was a significant difference between the spiritual well-being level of the patients who had less than high-school education and the ones with a bachelor's degree (*P* < 0.05). In terms of resilience, there were significant differences between all the levels of education (*P* < 0.001): the mean scores showed that higher levels of education correlate with better spiritual well-being and resilience.

The *t*-test results showed that there was a significant difference between the male and female patients in terms of spiritual well-being and resilience: the males had higher mean scores than the females (*P* < 0.001). Also, the *t*-test results showed that the married patients had significantly higher levels of spiritual well-being and resilience than the single patients (*P* < 0.001) (Table 2).

The subjects' resilience mean score was 54.84 ± 13.25; their spiritual well-being mean score was 73.22 ± 13.01. The results of Pearson's correlation coefficient test showed that there was a significant direct correlation between the participants' spiritual well-being and resilience: higher spiritual well-being scores correlated with higher resilience scores (*P* < 0.001) (Table 3).

As Table 4 shows, the correlation coefficient between religious well-being and resilience in the first step is 0.60. The square of the correlation coefficient which measures the predictive power of resilience based on the previous variable is 0.36, which remains 0.36 after moderation. The value of AR2 shows that religious well-being predicts 36% of the variance of resilience. B represents the non-standardized regression coefficients and *β* represents the standardized regression coefficients. A *β* of 1.25 means that one unit of change in religious well-being changes the variable of resilience by 1.25 units, whose statistical change is, considering the value of *t* at 7.78, significant at 0.05. With the addition of existential well-being to the model in the second step, the value of the moderated coefficient rises to 43%. This means that religious well-being and existential well-being significantly (*t* = 4.34) determine about 43% of the variance of resilience, and one unit of change in the standard deviation of existential and religious well-being changes resilience by 0.69 units.

## 4. Discussion

The results of the analysis of the relationship between the subjects' demographical variables on one hand and resilience and spiritual well-being on the other indicated that there is a significant correlation between education, marital status, age, gender, and the duration of the disease on one hand and resilience on the other. An increase in patients' age, academic status, and duration of the disease correlates with an increase in their resilience and spiritual well-being. The married patients reported higher levels of resilience and spiritual well-being. Furthermore, the male patients were found to have more spiritual well-being and resilience than the female patients. The difference in the degree of resilience between the male and female patients is consistent with the findings of an Iranian study on diabetic patients [48]; however, in another study on patients with cancer, the male and female patients do not differ in terms of resilience [49]. This discrepancy in the findings can be attributed to the differences between the natures of the diseases: psoriasis is a disease which affects an individual's appearance and, since women are more concerned about their appearances, they suffer from more stress.

According to a study in Iran, resilience correlates with academic degree [50], which is consistent with the results of the present study. In this regard, it seems that people with a higher academic status are equipped with more capabilities and skills to cope with stressful situations and thus can accept and fight diseases better. Moreover, a study in China reports that married people diagnosed with arthritis show more resilience than unmarried patients [51]. As for the correlation between resilience and age, a study in the United States finds more resilience in older patients suffering from multiple sclerosis [52], which may be due to the fact that, with an increase in age, people accept the reality of a disease more easily, learn how to cope with a disease, and perhaps pay less attention to their appearances.

The analysis of the results of the present study showed that spiritual well-being predicts 43% of the variance of resilience in patients with psoriasis. These findings show that individuals with higher levels of spiritual, existential, and religious well-being are more resilient, as they possess more personal strength, trust their intuition, have more positive emotions, and have more control over their personal matters. In this regard, the results of a study in India show that more resilient individuals report more perceived social support and are healthier [53]. Likewise, a study in Iran reports a positive, significant relationship between resilience and spiritual well-being [54]. The results of a study in the United States show that spiritual variables have a significant impact on resilience in patients with chronic diseases [55]. According to another study conducted in the United States, spirituality correlates with resilience and health in children suffering from traumatic experiences [56].

In a study in Iran, 51% of the variance of resilience of female adolescent students is predictable through their spiritual well-being [57]. This amount is more than twice the level of variance observed in the present study, whose discrepancy can be attributed to the fact that the participants in the present study were both male and female, of different age groups, and affected by a disease. On the other hand, according to a study on patients with chronic diseases, there is not a significant relationship between spirituality on one hand and resilience and quality of life on the other, which is contrary to the findings of the present study [58]. Considering the major role of religious well-being in spirituality and the stronger religious beliefs in Iran, this discrepancy can be explained through differences between cultures and perceptions of spirituality in social situations.

By creating an optimistic attitude towards the potency of non-materialistic forces, spirituality results in an increase in one's ability to bear hardships, which in turn results in greater resilience [59]. In addition, spirituality increases an individual's cognitive flexibility and resilience by inducing him/her to accept and come to terms with difficult situations [60, 61]. Spirituality, as a valuable cultural variable, correlates with health-related variables and quality of life in chronic diseases, produces more resilience, and increases patients' chances of recovery [55]. Patients with higher levels of spiritual well-being are more successful in coping with and adapting to the difficult conditions caused by chronic diseases [62]. Patients with psoriasis who possess better spiritual well-being are better able to adapt to their condition and, therefore, report higher levels of resilience and stress management skills.

One of the limitations of the present study is that it does not address such factors as the intensity of the disease and other psychological disorders which might affect the relationship between spiritual well-being and resilience. Also, the patients' self-reports as to not suffering from any psychological problems were considered to be true, while they might have had a psychological problem that they were not aware of or did not desire to reveal. Therefore, it is recommended that future studies further explore patient's psychological conditions.

## 5. Conclusion

The results of the present study show a positive, significant correlation between spiritual well-being and resilience in patients diagnosed with psoriasis: increased resilience correlates with higher degrees of spiritual well-being in the domains of existential well-being and religious well-being. The present study is one of the few studies which address the variables of spiritual well-being and resilience in patients with a chronic skin disease. Considering the chronic nature of psoriasis, it is recommended that care-providers include measures to improve spiritual well-being and resilience skills in the care plans designed for patients with psoriasis. As religious well-being is a significant factor in spirituality, it appears that interventions in this area can enhance patients' resilience. An increase in either of the two variables of spiritual well-being and resilience results in an increase in the other. An identification of the causal relationship between the two factors requires more research. Accordingly, it is suggested that resilience- and spirituality-based interventions be applied for patients with chronic diseases.

## Figures and Tables

**Table 1:** The participants' demographic characteristics (*N* = 150).

Variable	Group	Frequency	Percentage
Age	Under 30	56	37.3
	30–40	47	31.3
	Above 40	47	31.3

Duration of disease	Under 4 yrs.	47	31.3
	4–7 yrs.	35	23.3
	Above 7 yrs.	68	45.3

Marital status	Married	90	60.0
	Single	60	40.0

Education	High school and below	34	22.7
	Diploma	50	33.3
	Bachelor	49	32.7
	Master	17	11.3

Gender	Male	75	50
	Female	75	50

**Table 2:** Correlations between the participants' spiritual well-being and resilience with their demographic characteristics (*N* = 150).

Variable	Group	Spiritual well-being		Resilience		Result of ANOVA	
		Mean	Sd	Mean	Sd	Spiritual well-being	Resiliency
Age	Under 30	57.3	23.3	49.8	1.6	*F* = 13.2 *P* < 0.001	*F* = 5.3 *P* < 0.001
	30–40	75.8	19.9	57.3	1.8		
	Above 40	89.4	21.0	58.4	1.8		

Duration of disease	Under 4 yrs.	61.7	22.3	50.2	14.3	*F* = 28.7 *P* < 0.001	*F* = 7.1 *P* < 0.001
	4–7 yrs.	68.2	18.8	54.5	9.7		
	Above 7 yrs.	83.8	23.5	58.2	13.2		

Marital status	Single	57.9	23.9	48.3	13.1	*t* = 6.9 *P* < 0.001	*t* = 5.3 *P* < 0.001
	Married	83.4	20.7	59.1	11.4		

Education	High school and below	61.2	27.9	40.8	1.5	*F* = 5.2 *P* < 0.05	*F* = 55.9 *P* < 0.001
	Diploma	71.3	18.2	51.1	1.2		
	Bachelor	82.2	26.8	63.9	1.3		
	Master	76.7	24.3	67.2	2.5		

Gender	Male	52.0	14.4	52.1	11.0	*t* = 18.7 *P* < 0.001	*t* = 2.5 *P* < 0.05
	Female	94.3	13.1	57.5	14.6		

*P* < 0.05.

**Table 3:** Correlations between spiritual well-being and resilience (*N* = 150).

Variable	1	2
Resilience	–	
Spiritual well-being	0.46^*∗*^	–

^*∗*^Level of significance for all the values is *P* < 0.001.

**Table 4:** A precis of the regression analysis results for predicting resilience based on spiritual well-being (*N* = 150).

Variable	R	R2	AR2	B	Β	T
Constant	0.60	0.36	0.36	31.63	1.25	12.47^*∗*^
Religious well-being	0.66	0.44	0.43	1.27	0.69	7.78^*∗*^
Religious well-being and existential well-being				0.75		4.34^*∗*^

^*∗*^Level of significance for all the values is *P* < 0.001.

## Data Availability

All data used to support the findings of this study are included within the article. Moreover, the SPSS data used to support the findings of this study are available from the corresponding author upon request.

## References

[1] Eyerich S., Zielinski C. E. (2014). Defining Th-cell subsets in a classical and tissue-specific manner: examples from the skin. *European Journal of Immunology*.

[2] Ly K., Beck K. M., Smith M. P., Thibodeaux Q., Bhutani T. (2019). Diagnosis and screening of patients with generalized pustular psoriasis. *Psoriasis: Targets and Therapy*.

[3] Danielsen K., Olsen A. O., Wilsgaard T., Furberg A.-S. (2013). Is the prevalence of psoriasis increasing? A 30-year follow-up of a population-based cohort. *British Journal of Dermatology*.

[4] Parisi R., Symmons D. P. M., Griffiths C. E. M., Ashcroft D. M. (2013). Global epidemiology of psoriasis: a systematic review of incidence and prevalence. *Journal of Investigative Dermatology*.

[5] Ding X., Wang T., Shen Y. (2012). Prevalence of psoriasis in China: a population-based study in six cities. *European Journal of Dermatology*.

[6] Mohd Affandi A., Khan I., Ngah Saaya N. (2018). Epidemiology and clinical features of adult patients with psoriasis in Malaysia: 10-year review from the Malaysian psoriasis registry (2007-2016). *Dermatology Research and Practice*.

[7] Kubota K., Kamijima Y., Sato T. (2015). Epidemiology of psoriasis and palmoplantar pustulosis: a nationwide study using the Japanese national claims database. *BMJ Open*.

[8] Huerta C., Rivero E., Rodríguez L. A. G. (2007). Incidence and risk factors for psoriasis in the general population. *Archives of Dermatology*.

[9] Barrea L., Nappi F., Di Somma C. (2016). Environmental risk factors in psoriasis: the point of view of the nutritionist. *International Journal of Environmental Research and Public Health*.

[10] Leovigildo É. S., David R. A. R., Mendes A. S. (2016). Stress level of people with psoriasis at a public hospital. *Anais brasileiros de dermatologia*.

[11] Nazik H., Nazik S., Gul F. (2017). Body image, self-esteem, and quality of life in patients with psoriasis. *Indian Dermatology Online Journal*.

[12] Griffiths C., Barker J., Bleiker T., Chalmers R., Creamer D. (2017). *Rook’s Textbook of Dermatology*.

[13] Feldman S. R., Malakouti M., Koo J. Y. (2014). Social impact of the burden of psoriasis: effects on patients and practice. *Dermatology Online Journal*.

[14] Picardi A., Pasquini P. (2007). Toward a biopsychosocial approach to skin diseases. *Psychological Factors Affecting Medical Conditions*.

[15] Xhaja A., Shkodrani E., Frangaj S., Kuneshka L., Vasili E. (2014). An epidemiological study on trigger factors and quality of life in psoriatic patients. *Materia Socio Medica*.

[16] Tampa M., Sarbu M.-I., Mitran M.-I., Mitran C.-I., Matei C., Georgescu S.-R. (2018). The pathophysiological mechanisms and the quest for biomarkers in psoriasis, a stress-related skin disease. *Hindawi Disease Markers*.

[17] Naderi Azad S. (2018). Psoriasis: treating the skin and mind. *University of Toronto Medical Journal, Brain Health*.

[18] Shenefelt P., Shenefelt D. (2014). Spiritual and religious aspects of skin and skin disorders. *Psychology Research and Behavior Management*.

[19] Finan P. H., Zautra A. J., Wershba R., Contrada R. (2011). The dynamics of emotion in adaptation to stress. *The Handbook of Stress Science: Biology, Psychology, and Health. 16. IndieBound*.

[20] Oji V., Hung L. C., Abbasgholizadeh R., Terrell Hamilton F., Essien E. J., Nwulia E. (2017). Spiritual care may impact mental health and medication adherence in HIV+ populations. *HIV/AIDS-Research and Palliative Care*.

[21] Targ E. F., Levine E. G. (2002). The efficacy of a mind-body-spirit group for women with breast cancer: a randomized controlled trial. *General Hospital Psychiatry*.

[22] MacLean C. D., Susi B., Phifer N. (2003). Patient preference for physician discussion and practice of spirituality. *Journal of General Internal Medicine*.

[23] Gall T. L., Charbonneau C., Clarke N. H., Grant K., Joseph A., Shouldice L. (2005). Understanding the nature and role of spirituality in relation to coping and health: a conceptual framework. *Canadian Psychology/Psychologie Canadienne*.

[24] Oman D., Palautzian R. F., Park C. L. (2013). Defining religion and spirituality. *Handbook of the Psychology of Religion and Spirituality*.

[25] Tassel-Matamua N. A., Frewin K. E. (2019). Psycho-spiritual transformation after an exceptional human experience. *Journal of Spirituality in Mental Health*.

[26] Rinkel M., Larsen K., Harrington C., Chun C. (2018). Effects of social work practice on practitioners’ spirituality. *Journal of Religion & Spirituality in Social Work: Social Thought*.

[27] Stoltzfus M. J., Green R. (2013). *Spirituality, Chronic Illness, and Healing: Unique Challenges and Opportunities. Chronic Illness, Spirituality, and Healing*.

[28] Lucette A., Ironson G., Pargament K. I., Krause N. (2016). Spirituality and religiousness are associated with fewer depressive symptoms in individuals with medical conditions. *Psychosomatics*.

[29] Corbett M., Lovell M., Siddall P. J. (2017). The role of spiritual factors in people living with chronic pain: a qualitative investigation. *Journal for the Study of Spirituality*.

[30] Vitorino L. M., Lucchetti G., Leão F. C., Vallada H., Peres M. F. P. (2018). The association between spirituality and religiousness and mental health. *Scientific Reports*.

[31] Mohammadi M., Alavi M., Bahrami M., Zandieh Z. (2017). Assessment of the relationship between spiritual and social health and the self-care ability of elderly people referred to community health centers. *Iranian Journal of Nursing and Midwifery Research*.

[32] Vallurupalli M., Lauderdale K., Balboni M. J. (2012). The role of spirituality and religious coping in the quality of life of patients with advanced cancer receiving palliative radiation therapy. *The Journal of Supportive Oncology*.

[33] Azarsa T., Davoodi A., Khorami Markani A., Gahramanian A., Vargaeei A. (2015). Spiritual wellbeing, attitude toward spiritual care and its relationship with spiritual care competence among critical care nurses. *Journal of Caring Sciences*.

[34] Yang E. J., Beck K. M., Liao W. (2018). Secukinumab in the treatment of psoriasis: patient selection and perspectives. *Psoriasis: Targets and Therapy*.

[35] Liu H., Zhang C., Ji Y., Yang L. (2018). Biological and psychological perspectives of resilience: is it possible to improve stress resistance?. *Frontiers in Human Neuroscience*.

[36] Elisei S., Sciarma T., Verdolini N., Anastasi S. (2013). Resilience and depressive disorders. *Psychiatria Danubina*.

[37] Rosenberg A. R., Syrjala K. L., Martin P. J. (2015). Resilience, health, and quality of life among long-term survivors of hematopoietic cell transplantation. *Cancer*.

[38] Kim G. M., Lim J. Y., Kim E. J., Park S. M. (2019). Resilience of patients with chronic diseases: a systematic review. *Health & Social Care in the Community*.

[39] Connor K. M., Davidson J. R. T. (2003). Development of a new resilience scale: the connor-davidson resilience scale (CD-RISC). *Depression and Anxiety*.

[40] Resnick B., Gwyther L. P., Roberto K. (2011). *Resilience in Aging Concepts Research and Outcomes*.

[41] Ferreira B. I. R. C., Abreu J. L. P. D. C., Dos Reis J. P. G., Figueiredo A. M. D. C. (2016). Psoriasis and associated psychiatric disorders: a systematic review on etiopathogenesis and clinical correlation. *The Journal of Clinical and Aesthetic Dermatology*.

[42] Mahmutovic J., Zukic M., Pasalic A., Brankovic S., Jaganjac A., Katana B. (2017). Correlation between quality of life and depression among persons suffering from psoriasis. *Medical Archives*.

[43] Smith L., Webber R., DeFrain J. (2013). Spiritual well-being and its relationship to resilience in young people: a mixed methods case study. *Sage Open*.

[44] Ellison C. W. (1983). Spiritual well-being: conceptualization and measurement. *Journal of Psychology and Theology*.

[45] Paloutzian R. F., Park C. L. (2014). *Handbook of the Psychology of Religion and Spirituality*.

[46] Allahbakhshian M., Jaffarpour M., Parvizy S., Haghani H. (2010). A survey on relationship between spiritual wellbeing and quality of life in multiple sclerosis patients. *Zahedan Journal of Research in Medical Sciences*.

[47] Samani S., Jokar B., Sahragard N. (2007). Effects of resilience on mental health and life satisfaction. *Iranian Journal of Psychiatry and Clinical Psychology*.

[48] Mazlom Bafroe N., Shams Esfand Abadi H., Jalali M., Afkhami Ardakani M., Dadgari A. (2015). The relationship between resilience and hardiness in patients with type 2 diabetes in yazd. *The Journal of Shahid Sadoughi University of Medical Sciences*.

[49] Nejat N., Whitehead L., Crowe M. (2017). The use of spirituality and religiosity in coping with colorectal cancer. *Contemporary Nurse*.

[50] Manning L., Ferris M., Narvaez Rosario C., Prues M., Bouchard L. (2019). Spiritual resilience: understanding the protection and promotion of well-being in the later life. *Journal of Religion, Spirituality & Aging*.

[51] Rojas M., Rodriguez Y., Pacheco Y. (2018). Resilience in women with autoimmune rheumatic diseases. *Joint Bone Spine*.

[52] Black R., Dorstyn D. (2015). A biopsychosocial model of resilience for multiple sclerosis. *Journal of Health Psychology*.

[53] Somasundaram R., Devamani K. (2016). A comparative study on resilience, perceived social support and hopelessness among cancer patients treated with curative and palliative care. *Indian Journal of Palliative Care*.

[54] Khosravi M., Nikmanesh Z. (2014). Relationship of spiritual intelligence with resilience and perceived stress. *Iranian Journal of Psychiatry and Behavioral Sciences*.

[55] Hunter-Hernández M., Costas-Muñíz R., Gany F. (2015). Missed opportunity: spirituality as a bridge to resilience in Latinos with cancer. *Journal of Religion and Health*.

[56] Brewer-Smyth K., Koenig H. G. (2014). Could spirituality and religion promote stress resilience in survivors of childhood trauma?. *Issues in Mental Health Nursing*.

[57] Dehghani F., Andishmand V. (2017). The relationship of religious orientation and spiritual health to resilience among high school sophomores in kerman. *Journal of Research on Religion & Health*.

[58] Vinaccia S., Quiceno J. M., Remor E. (2012). Resiliencia, percepción de enfermedad, creencias y afrontamiento espiritual-religioso en relación con la calidad de vida relacionada con la salud en enfermos crónicos colombianos. *Anales de Psicología/Annals of Psychology*.

[59] Souri H., Hasanirad T. (2011). Relationship between resilience, optimism and psychological well-being in students of medicine. *Procedia-Social and Behavioral Sciences*.

[60] Plexico L. W., Erath S., Shores H., Burrus E. (2019). Self-acceptance, resilience, coping and satisfaction of life in people who stutter. *Journal of Fluency Disorders*.

[61] Kopacz M. S., Silver E., Bossarte R. M. (2014). A position article for applying spirituality to suicide prevention. *Journal of Spirituality in Mental Health*.

[62] Reynolds N., Mrug S., Hensler M., Guion K., Madan-Swain A. (2014). Spiritual coping and adjustment in adolescents with chronic illness: a 2-year prospective study. *Journal of Pediatric Psychology*.

